# Simulations of Phage T7 Capsid Expansion Reveal the Role of Molecular Sterics on Dynamics

**DOI:** 10.3390/v12111273

**Published:** 2020-11-07

**Authors:** Paul C. Whitford, Wen Jiang, Philip Serwer

**Affiliations:** 1 Department of Physics, Northeastern University, Boston, MA 02115, USA; 2 Center for Theoretical Biological Physics, Northeastern University, Boston, MA 02115, USA; 3 Department of Biological Sciences, Markey Center for Structural Biology, Purdue University, West Lafayette, IN 47906, USA; jiang12@purdue.edu; 4 Department of Biochemistry and Structural Biology, The University of Texas Health Center, San Antonio, TX 78229-3900, USA; serwer@uthscsa.edu

**Keywords:** bacteriophage T7, disorder, DNA packaging, molecular dynamics simulation, protein dynamics

## Abstract

Molecular dynamics techniques provide numerous strategies for investigating biomolecular energetics, though quantitative analysis is often only accessible for relatively small (frequently monomeric) systems. To address this limit, we use simulations in combination with a simplified energetic model to study complex rearrangements in a large assembly. We use cryo-EM reconstructions to simulate the DNA packaging-associated 3 nm expansion of the protein shell of an initially assembled phage T7 capsid (called procapsid or capsid I). This is accompanied by a disorder–order transition and expansion-associated externalization displacement of the 420 N-terminal tails of the shell proteins. For the simulations, we use an all-atom structure-based model (1.07 million atoms), which is specifically designed to probe the influence of molecular sterics on dynamics. We find that the rate at which the N-terminal tails undergo translocation depends heavily on their position within hexons and pentons. Specifically, trans-shell displacements of the hexon E subunits are the most frequent and hexon A subunits are the least frequent. The simulations also implicate numerous tail translocation intermediates during tail translocation that involve topological traps, as well as sterically induced barriers. The presented study establishes a foundation for understanding the precise relationship between molecular structure and phage maturation.

## 1. Introduction

While a major objective in molecular biophysics research is to establish physical descriptions of energy-requiring, motor-like biological processes, distilling the complex nature of the dynamics is a continual challenge. For comparison, physical descriptions of macroscopic motors are based on well-controlled repetitive motions. For such systems, a deterministic view is appropriate to describe the energetics, for example, when determining the maximum efficiency of an ideal motor. However, for biochemical, nanometer-scale motors, the physical description is complicated by the intrinsic stochasticity of microscopic motion. Although cycles repeat, on average, details of each event vary due to Brownian noise that is introduced from thermal energy. This naturally leads to biomolecules exhibiting a significant degree of flexibility [1,2,3], where individual molecules have even been described as “forever kicking and screaming” [4].

While flexibility of motor components helps define biology, this flexibility also introduces difficulties in the analysis of dynamics. However, these challenges can be reduced when the motor components are assembled from many identical molecules that arrange symmetrically. In those cases, symmetry can amplify the data available for determining structures, which are necessary for the application of molecular simulations. The nucleic acid-containing protein shells (capsids) of viruses represent one such class of systems [5,6,7]. Analysis of viral capsid structure, by use of both X-ray crystallography and cryo-electron microscopy (cryo-EM), was a major contribution of the laboratory of Dr. Michael Rossmann [5,8,9,10], to whom the current symposium is dedicated. Here, we build upon his seminal contributions to begin the detailed analysis of dynamics in these complex systems.

Historically, our philosophical background begins with the projection that new physical principles might be discovered in biology [11]. Within biology, countless challenging physical questions have surfaced through the years. For example, conventional biochemical thought (i.e., Levinthal’s paradox [12]) initially made it difficult to understand the origins of rapid protein folding. However, the adoption of condensed matter physics principles enabled systematic investigation into the many energetic factors that influence folding dynamics [13,14,15]. Another outstanding challenge is how to understand the duality of power stroke vs. thermal ratchet mechanisms (reviewed in [16,17]). One could also look to larger length scales and inquire about the organizational principles of chromosomes [18,19], or seek to understand the properties that give rise to membrane-less organelles in the cell [20]. Each of these biological phenomena pose distinct challenges, where new physical principles have frequently been proposed and tested, in order to establish precise quantitative insights.

**Figure 1:**
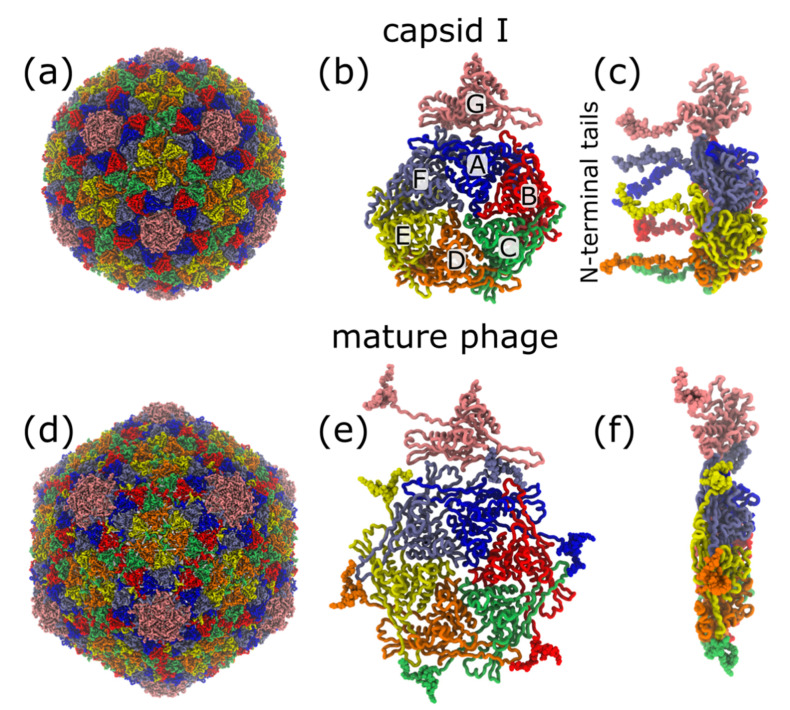
Expansion during phage maturation involves ordering of the N-terminal tails of gp10. (**a**) Cryo-EM structure of T7 capsid I (PDB:3J7V) [23]. (**b**) Exterior-shell view of asymmetric subunit in capsid I conformation. Monomer positions labelled A–F. (**c**) Side view of panel b. Modeled gp10 N-terminal tails (vdW representation) are on the interior of the shell. (**d**) Cryo-EM structure of the mature phage (PDB:3J7X) [23]. (**e**) Exterior-shell view of asymmetric subunit. (**f**) Side view of panel e. All structural representations are generated with VMD [27].

Here, we study phage T7 capsids and explore the structural dynamics (Figure 1) that accompany packaging of DNA (reviewed in [21,22]). Starting with a DNA-free capsid I, packaging is associated with (1) activity of a DNA packaging-driving ATPase and (2) expansion and structural change of the capsid, ultimately to form a structure called capsid II that, with minor changes, becomes the capsid of the phage (Figure 1) [22,23,24]. When describing the capsid, the proteins are labeled gp (gene product), followed by gene number [25,26]. The capsid I, capsid II and phage shells are composed of 420 gp10 proteins, and capsid structures have been previously determined by cryo-EM to 4.6, 3.5 and 3.6 Å resolution, respectively [23].

In the current study, we focus simulations on the intermediate states of the disordered 23 amino acid, N-terminal gp10 tail (Figure 1). This tail moves from the inner surface of capsid I to the outer surface when capsid I expands to capsid II and phage capsid [23]. To elucidate the influence of molecular sterics on capsid maturation, we apply simulations to directly study the timing of N-terminal tail translocation and shell expansion. This shows how computational and theoretical techniques can now be leveraged to study global conformational motions of a complete gp10 capsid shell.

In the simulations, we apply a “structure-based” model [28], in which all non-hydrogen atoms are explicitly represented (1.07 million atoms). These models have been used extensively to study global rearrangements in other large-scale assemblies, including the ribosome [29] and HIV capsid [30]. While coarse-grained variants of this model have been applied to investigate structural fluctuations of the HK97 capsid shell [31], to the best of our knowledge, there have not been simulations reported of spontaneous (i.e., without the use of targeting methods) expansion events that include ordering of the terminal tails. Accordingly, the presented simulations demonstrate the feasibility of all-atom molecular dynamics simulations to characterize coordinated rearrangements within a complete capsid. These simulations additionally reveal a complex interplay of expansion, steric interactions and order–disorder phenomena that accompanies the maturation process. Taken together, these observations suggest new strategies by which biochemical and structural techniques may obtain more precise insights into DNA packaging-driven dynamics.

## 2. Materials and Methods

### 2.1. Structure of Phage T7 and its Capsid I and Capsid II

The structures of phage T7, capsid I (procapsid) and capsid II are described in reference [23]. Since the N-terminal tails (residues 1–23) were not resolved in the capsid I state, we generated disordered initial conformations. To generate a random ensemble of disordered conformations for each of the 420 tails, a high-temperature simulation (reduced temperature of 1) was initiated from the capsid I conformation, with non-tail residues constrained to their capsid I positions. Specifically, an all-atom structure-based model [28,30] was defined for the capsid I structure, while all non-tail atoms were constrained to their initial positions. By performing a high-temperature simulation, any bias of the force field is negligible, such that the tails sample a fully disordered range of conformations. Four randomly selected disordered conformations were used to initialize simulations of capsid expansion.

### 2.2. Force Field Description

For the presented simulations, an all-atom structure-based “SMOG” force field [28,30] was defined based on the structure of the mature phage gp10 shell (PDB:3J7X) [23]. As described elsewhere [30], all non-bonded atomic contacts (identified with the Shadow algorithm [32]) and flexible dihedral interactions are set to specifically stabilize the expanded conformation. The interaction distance of each contact was scaled by 0.95, similar to applications of this model to the ribosome [33]. The purpose of this modification is to avoid artificial expansion beyond that found in the mature phage conformation. This ensures that the free energy as a function of the radius of gyration has a minimum that coincides with the value found in the mature phage structure. The only differences from the previous implementation of the model are in the bonded interactions. The bond lengths and angles are assigned the values found in the Amber ff03 force field [34], and rigid dihedral angles (e.g., hybridized, planar) are assigned a cosine potential of periodicity 2 with minima at 0 and 180 degrees.

### 2.3. Simulation Details

All simulations were performed using the GROMACS software package [35,36] (v5.1.4), and the force field was generated using SMOG 2 [30]. Each simulation was performed at a reduced temperature of 0.582 (70 in GROMACS units), which is roughly 0.6–0.7 times the folding–unfolding temperature of a typical single-domain protein with this model [37]. Further, since physiological temperature for a given organism is generally ~0.7 of the folding temperature of its constituent proteins, this temperature range tends to yield molecular fluctuations that are representative of cellular conditions [37]. Constant temperature was maintained via Langevin Dynamics protocols. A total of 20 simulations were performed for 1.4–3.0 × 10^8^ timesteps of duration 0.002 reduced units (3.4 × 10^9^ timesteps, in aggregate). The duration of each simulation was dependent on available computational resources and shared-resource scheduler policies. Each simulation was performed on 112 compute cores, where an aggregate ~1.6 million core-hours was required to perform the set of simulations. To ensure that the results were not dependent on the precise initial conformation, four different randomly selected initial conformations were used, where five simulations were performed for each (20 total simulations). Each simulation was initialized with randomized velocities and Langevin noise. Based on a recent comparison of diffusion coefficients in a SMOG model and an all-atom explicit-solvent model [38], one may estimate the effective simulated time to be approximately 250–500 microseconds, for each simulation.

## 3. Results

### 3.1. Molecular Dynamics Simulation of Spontaneous Capsid Expansion

**Figure 2:**
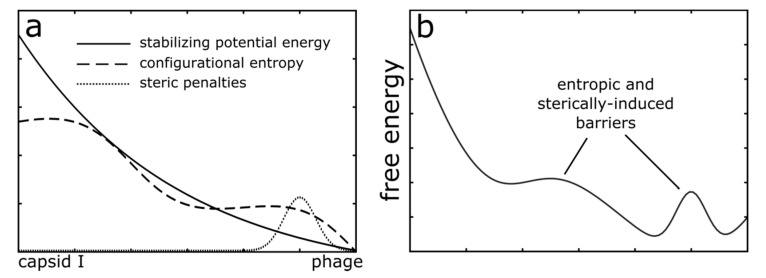
Schematic depiction of the modeled energetics. (**a**) In the current study, MD simulations utilized an all-atom structure-based model [28,30], where the mature phage conformation was defined as the minimum potential energy conformation. Since all stabilizing interactions are defined based on the mature phage conformation, the potential energy is “downhill” in character (solid line). Despite this simplified representation, configurational entropy (large dashed line) and sterically-induced penalties (small dashed line) can arise from the molecular structure and flexibility. (**b**) Together, the potential energy, sterics and configurational entropy can lead to a complex free-energy landscape that has many barriers.

Since our long-term objective is to understand how individual molecular interactions facilitate capsid assembly and maturation, we will begin by asking whether steric effects can limit expansion-associated transitions of the N-terminal tails of gp10. For this, we initiated simulations from the compact capsid I conformation (Figure 1a). Consistent with the lack of density for the N-terminal tails in the cryo-EM reconstruction of the capsid I structure, the N-terminal tails were assigned disordered conformations (see Methods). In each simulation, the system was allowed to move under the influence of a mature phage capsid-specific force field. That is, we applied an all-atom structure-based model [28,30], where every non-bonded interaction in the mature phage conformation was defined to be stabilizing. Based on this construction, the overall potential energy landscape may be considered “downhill” (Figure 2a) in the direction of the mature conformation. Since the expansion process is known to be driven by DNA packaging, the potential energy in this model is intended to describe the *effective energetics* [39,40,41] of the system. Accordingly, the net effect of DNA packaging is to favor the expanded form, which is implicitly embedded in this model by defining the mature form to be the potential energy minimum. Even though DNA and solvent are not explicitly represented, since the model explicitly represents all non-hydrogen atoms in the capsid (1.07 million atoms), this simplified energetic representation can be used to isolate specific types of factors that can give rise to free-energy barriers. For example, this model can identify barriers that stem from (1) the imperfect structural complementarity of the tail and corresponding cavity, or (2) confinement effects that impose sharp changes in the configurational entropy. Together, these factors can result in a rugged, expansion-favoring free-energy landscape (Figure 2b).

**Figure 3:**
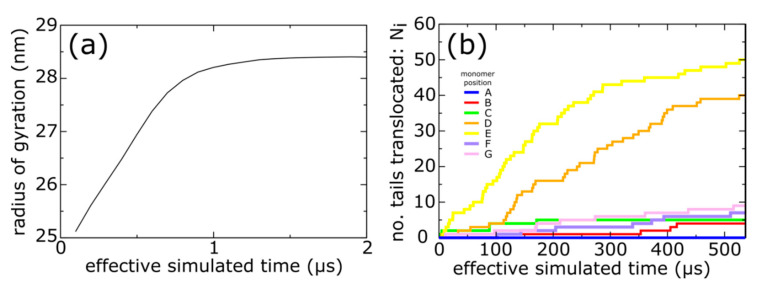
Simulations of spontaneous capsid expansion events reveal that N-terminal tail translocation is position-dependent. (**a**) Representative time trace of a simulation with an all-atom structure-based model shows that, based on the radius of gyration, the capsid expands during the first few microseconds. (**b**) The N-terminal tails translocate on longer timescales (hundreds of microseconds). A tail is considered to have completed translocation once 80% of the phage-specific tail contacts are formed. The same simulation is shown as in panel a.

Even though our model explicitly represents every non-hydrogen atom (Figure 1), the simplicity of the structure-based force field allows us to simulate spontaneous (i.e., non-targeted) expansion events (Video S1). During each simulation, the radius of gyration increases from its capsid I value of ~25 nm, to ~28.5 nm, as the system approaches the mature phage conformation (Figure 3a). If neither sterics nor configurational entropy limit expansion, then simulations with this model would lead to rapid translocation of the N-terminal tails, since the energetics explicitly favor their movement to the shell exterior.

In contrast, we find that the kinetics of trans-shell displacements depend strongly on the location of the gp10 monomer. To monitor the progress of each tail towards the phage conformation, we calculated the fraction of mature-phage contacts of the N-terminal tail (Ala2-Leu25; residue 1 was not resolved in any structure) that are formed as a function of time for each chain: *Q*_i_ (i = A−F forming the hexon and G is a subunit of the penton). A contact is defined as being “formed" if the distance between the contacting atom pair is less than 1.5 times their distance in the mature phage conformation. The use of “native” (i.e., mature-phage) contacts is motivated by its demonstrated utility for describing protein folding [42]. Based on *Q*_i_, we then define a chain as having completed shell translocation when at least 80% of the mature-phage-specific intermolecular tail contacts were formed. As a note, since simulations are always performed at finite temperatures, even a fully-folded protein will typically only have 70–90% of the native contacts formed at any given time [28]. In a representative trajectory (Figure 3b), the E monomers were associated with the largest number of translocated tails (*N*_E_ > 50), and the D position exhibited nearly as many events (*N*_D_ ~ 40). In stark contrast, we find that shell translocation was less frequent for monomers A–C, F and G. The least likely tails to translocate were those of the A monomers, which lacked any translocation events on the simulated timescale.

With regard to simulated times, the overall expansion process occurred on the microsecond scale (Figure 3a), whereas tail translocation typically required hundreds of microseconds (Figure 3b). Accordingly, the overall picture illustrated by this simulation is that capsid maturation involves an initial global expansion of the shell, followed by trans-shell displacement and ordering of the N-terminal tails. However, due to the simplified character of the model, one should be careful to not overinterpret these timescales. That is, rather than modeling DNA packaging as a time-dependent effect that is proportional to the fraction of packaged DNA, our model simply describes the mature phage as the global potential energy minimum. Accordingly, in solution, T7 expansion will occur on longer timescales that depend on the rate of DNA packaging.

### 3.2. Simulations Implicate Hierarchical Timescales for Trans-Shell Displacements

Inspecting individual simulations, as in Figure 3, can provide anecdotal suggestions into the character of a system. However, biomolecular processes are intrinsically stochastic [43]. Thus, it is necessary to consider statistical properties. To this end, we performed 20 independent simulations of the expansion process, each initialized with randomized velocities (see methods), and then analyzed tail translocation events across this ensemble. The total number of each simulated tail that successfully underwent a trans-shell rearrangement is given in Table 1.

**Figure 4:**
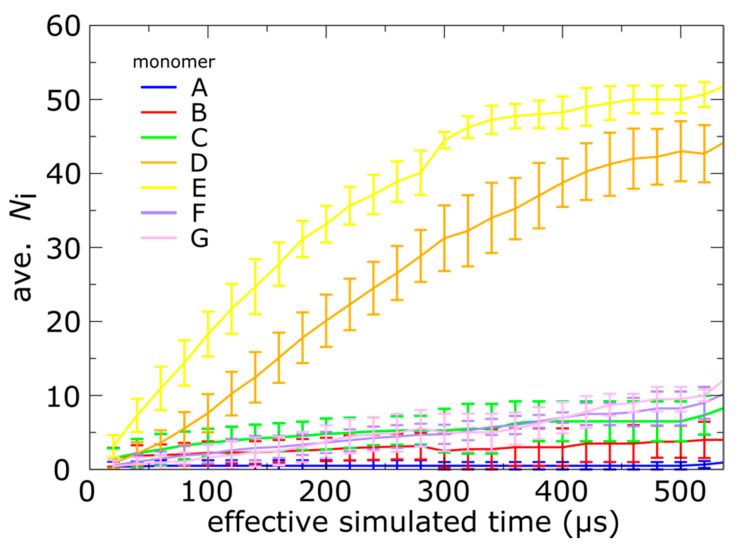
Position-dependent N-terminal dynamics is robust. Average number of translocated tails, *N*_i_, as a function of simulated time, where the average is taken over 20 independent simulations. Error bars represent one standard deviation, and monomers A–G are shown in different colors. Consistent with the representative trace in Figure 3b, monomers D and E are consistently faster than the remaining tails. Further, monomer A is consistently slowest, and it rarely reaches the exterior of the capsid shell in the simulations.

Consistent with the representative time traces in Figure 3b, the tails of monomers E and D were found to undergo trans-shell displacements most frequently, whereas tails B, C, F and G appear to be slower and translocate on comparable timescales (Figure 4). We found that not a single simulation yielded more than two translocated tails in the A position, which is also consistent with the slow dynamics of the A monomers. To identify whether the timescale differences are statistically significant, we also calculated the standard deviation as a function of time (error bars in Figure 4). Consistent with the stochasticity of biomolecular events, the standard deviation is non-negligible, and it ranges from ~1 to 5 tails. However, the differences between the D and E positions can exceed 10 tails, which shows that the position-dependence of the kinetics is preserved across the set of simulations. For monomers B, C, F and G, there is substantial overlap in the sampled *N*_i_ values, indicating that these chains translocate on similar timescales. However, monomer A is consistently the least likely to reach the phage conformation, even when the uncertainty is taken into account.

**Figure 5:**
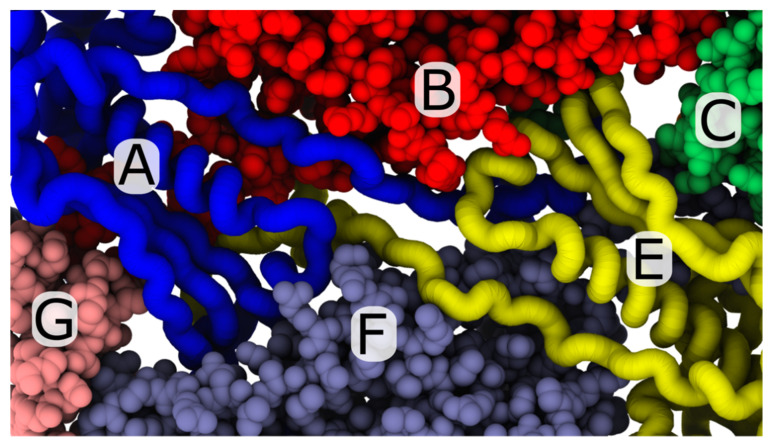
View of the trans-shell cavity associated with A- and E-monomer tails. In the mature phage conformation, the A (blue) and E monomers (yellow) adopt a nearly-rotationally-symmetric arrangement, where the N-terminal tails are positioned between B and F monomers. The perspective shown is from the interior of the capsid shell. Despite the fact that the A and E monomers share a common trans-shell cavity, the A monomer is far less likely to undergo trans-shell displacements in the simulations. A possible explanation of the asymmetric dynamics may be that the A monomer is adjacent to a penton (monomer G), while the E monomer is associated with a hexon (monomer C). Differences in the dynamics of expansion around the pentons and hexons have the potential to alter the positions of each N-terminal tail, which may then influence the dynamics of trans-shell displacements.

The difference in timescales between E and A monomers is surprising, because they share a common cavity (Figure 5). To better understand this observation, we calculated the average time for monomers to reach the exterior shell; the average only included successful crossing events. That is, we only considered the 10 and 845 successful trans-shell displacements of A and E monomers. While A monomers were less likely to reach the exterior of the shell, the successful events were found to occur on shorter timescales (1/10 the time) than successful E-monomer transitions. Since only successful events were considered, this differential timescale cannot be used to infer a rate. Instead, one should interpret it as suggesting the A monomers occasionally reach the exterior rapidly, but conformational changes in the capsid impede motion of tails that do not successfully cross in the initial stage of the simulation.

In summary, we find that translocation of the D- and E-monomer tails is frequent, while B-, C-, F- and G-monomer translocation appears to be substantially slower. Further, it appears that translocation of the A-monomer tails is impeded by prior passage of E-monomer tails. This apparent hierarchy of timescales is caused by differences in the monomer-proximal steric composition, which changes as the capsid expands. As described below, the subtle differences in local steric composition can also give rise to numerous intermediate conformations during maturation.

### 3.3. Capsid Shell Sterics Lead to Multiple Intermediates during N-tail Translocation

**Figure 6:**
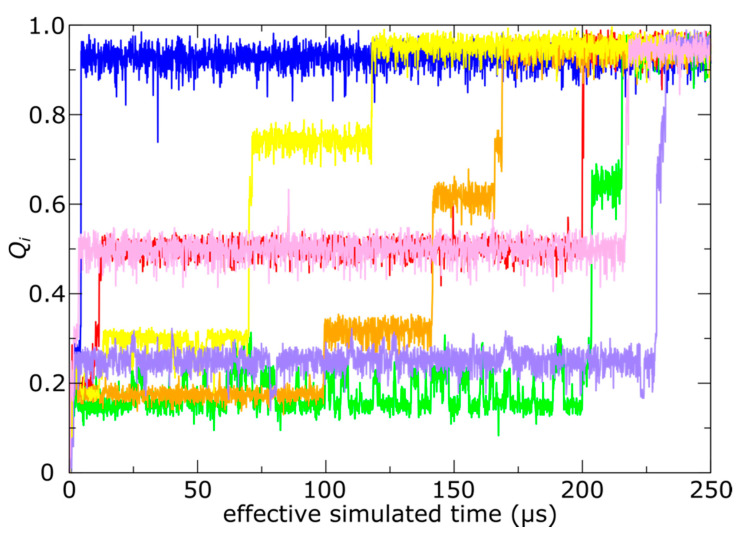
N-terminal tails sample intermediate ensembles. During trans-shell displacements, many tails transiently adopt relatively stable conformations in which only some of the phage-specific contacts are formed. Time traces show the fraction of phage-specific contacts formed (*Q*_i_) for representative monomers at each position (1, of 60, per capsid). The color of each trace signifies a different monomer position, where color assignments are consistent with Figure 3 and Figure 4. With the exception of the A (blue) and F (purple) monomers, all other positions display clear pauses at intermediate values of *Q*_i_.

In addition to facilitating position-dependent timescales, the steric composition of the capsid shell also leads to the adoption of various intermediate ensembles (intermediates) during N-terminal tail translocation. To identify intermediates, we inspected the number of mature phage-specific contacts that are formed, as a function of time. As described above, we considered the fraction of these contacts that are formed between the N-terminal tail and the capsid shell for each frame: *Q*_i_, where *i* denotes the monomer position. A value of zero indicates that the tail is on the interior of the capsid shell, whereas a value of 1 indicates that the tail has fully translocated and adopted its mature phage conformation. For illustrative purposes, we show *Q*_i_ for individual monomer tails in a single simulation (Figure 6). These representative time traces have plateaus, each of which reveals the presence of a relatively stable intermediate conformation during trans-shell displacements of the tail. As described below, these intermediates may be attributed to the dense steric environment of the shell.

**Figure 7:**
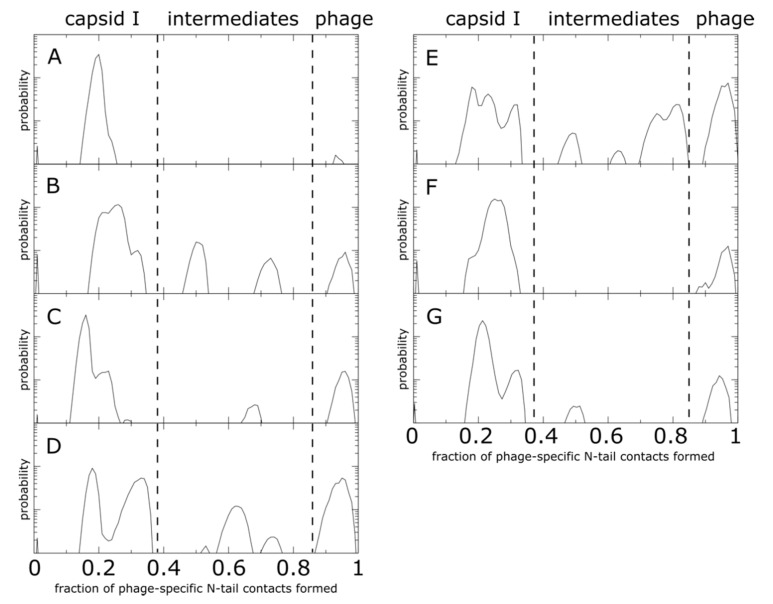
N-terminal tail intermediate populations during shell translocation. Each distribution represents the probability as a function of the fraction of mature-phage-specific tail–shell contacts formed (*Q*_i_), averaged over all monomers of the same position. The corresponding monomer position (**A**–**G**) is listed in the top left of each plot. Distributions were calculated from 20 independent simulations. Probability is shown on a log-linear plot, where the y axis spans three orders of magnitude. For all monomers, there are pronounced peaks at low values of *Q*_i_, consistent with the presence of an initial relaxation stage during which the tail samples conformations on the inner surface of the shell. There are also peaks at high *Q*_i_ values, which signify that tails in each position do reach the exterior surface of the shell. In addition to the endpoints, monomers B–E have peaks at intermediate values of *Q*_i_. This shows that the local steric environment introduced by each cavity can impede translocation at various points during the trans-shell displacement. Interestingly, there is no common pattern to the positions of the intermediate peaks, which further illustrates how differences in the steric composition of each cavity can lead to position-specific tail dynamics.

As done for our analysis of kinetics, we evaluated the statistics of tail dynamics. To describe the distribution of sampled conformations, we calculated the probability as a function of *Q*_i_ for each monomer position (Figure 7). We find that the distributions for monomers A and F only display dominant peaks at high (exterior positions) and low (interior positions) values of *Q*_i_. In contrast, monomers B–E and G all display peaks at intermediate values of *Q*_i_, indicating that these positions are likely to transiently halt progress towards the phage conformation. As with the above arguments, these features arise from the imperfect spatial complementarity of gp10 monomers. One may find examples of similar behavior in other systems, for example, where structure-based models have predicted a range of sterically-induced intermediate conformations of tRNA on the ribosome [29], even when the energetic direction is downhill.

Inspecting the structural properties of intermediate snapshots can highlight steric and topological features that impede capsid maturation in the simulations. For this discussion, we will consider the B monomer and associated cavity, since the B monomer exhibited two clear intermediate peaks (Figure 7), as well as relatively slow trans-shell displacement kinetics (Figure 4). Unlike the other cavities, the B-monomer cavity is associated with N-terminal tails that are from two monomers of the same position within the asymmetric subunit, which arrange around the 2-fold axis of the capsid. Here, we will consider structural snapshots for which *Q*_B_ is approximately 0.3, 0.5, 0.7 and 1.0.

**Figure 8:**
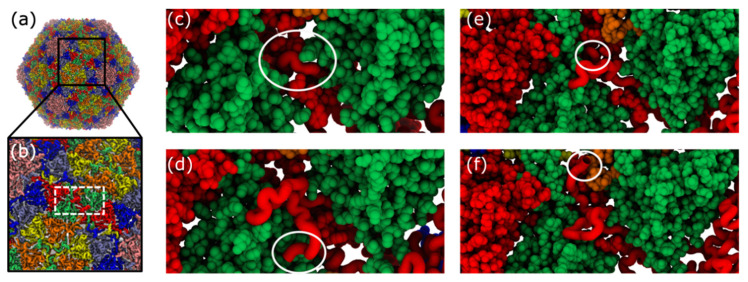
Structural representation of trans-shell displacement intermediates. During trans-shell displacement of the B monomer, various intermediate conformations are sampled (Figure 7). (**a**) Structure of mature phage structure. (**b**) Close-up view of shell exterior, centered about the B monomer cavity (white dashed box). (**c**) During B-monomer (red) translocation, the tail initially (*Q*_i_ ~ 0.3) forms a tight turn (circled). (**d**) On occasion, after reaching the exterior surface, only a low number of phage-specific contacts are formed (*Q*_i_ ~ 0.5), where the tail adopts a twisted conformation (circled), relative to the phage conformation (panel d). (**e**) In many simulations, the B monomer tail region is displaced through the shell, except for the final few terminal residues (circled). These terminal residues remain trapped within the dense steric environment imposed by the cavity. (**f**) Phage conformation of the B monomer tail. Note, in all images, the B monomer of interest is shown in tube representation, while the other B monomer associated with the same cavity is shown in vdW representation.

When *Q*_B_ ~ 0.3, the majority of the N-terminal tail is on the interior of the capsid shell, while a sharp loop-like arrangement has entered the trans-shell cavity (Figure 8c). Interestingly, a “loop-first” mode of cavity passage is reminiscent of the “slip-knot” mechanism seen during the folding of knotted proteins [44]. In those systems, it has been shown that the slip-knot mechanism is entropically favored over threading the terminal residue first, as the latter would require an abrupt reduction in configuration entropy. Based on this, we anticipate that slip-knotting will be a robust feature of trans-shell displacements in T7, irrespective of simulation details.

For larger values of *Q*_B_ (0.5 and 0.7), the tail is nominally on the exterior of the capsid shell. However, when *Q*_B_ ~ 0.5 (Figure 8d), the tail appears twisted, relative to the phage conformation (Figure 8f). While this is a relatively minor difference, in terms of atomic distances, the dense network of steric interactions limits the mobility of the tail, resulting in slow reorganization to the phage conformation. Interestingly, this phenomenon, where the order of native-contact formation introduces energetic traps, is similar to the observation of “topological frustration” in protein folding [45]. For *Q*_B_ ~ 0.7, the N-terminal tail is confined by the complementary B-monomer with which it shares the cavity (Figure 8e). These conformations may result from an incomplete slip-knot displacement of the N-terminal loop. Again, the densely packed environment introduces substantial steric obstacles that can significantly reduce the rate of adopting the phage conformation. Drawing on analogies with protein folding, it would appear that this caging effect may introduce glassy-like dynamics [14], despite the fact that the model is energetically downhill.

The presented simulations illustrate how molecular sterics and configurational entropy may contribute to the order and timing of N-terminal tail dynamics during phage maturation. This helps establish a baseline description, which opens a range of new areas of investigation. Building upon the simplified models described here, one could dissect the relative contributions of individual steric factors by introducing perturbations and comparing kinetics/mechanisms. As a motivation for such efforts, similar perturbative strategies have been extensively applied to reveal the influence of structure on dynamics in the ribosome [29]. With the demonstrated complexity of capsid maturation, there are now many variations to consider, in order to delineate the precise contributions of intermolecular interactions on large-scale collective dynamics.

## 4. Discussion

### 4.1. Non-Uniformity of Subunit Structure during Expansion

Although quasi-equivalence has been the foundation for thinking about the mature structure of virus capsids [6,7,8], non-equivalence of expansion intermediates emerges from the simulations performed here. This non-equivalence is derived from sterics-promoted, position-dependent heterogeneous kinetics. In capsid I, the initially-skewed hexon arrangement later adopts a nearly 6-fold symmetric structure in the mature phage. This subtle asymmetry may contribute to a large variability in the expansion dynamics of the various gp10 subunits. Thus, the intermediates are expected to have a structure with less equivalence than the mature structure.

Furthermore, the possibility exists that the non-equivalent dynamics of the tails are, in some conditions, a source of more global variability. For example, in the case of DNA packaging, DNA interaction with the slow tails, if it occurred before expansion completion, might cause a packaged DNA length-dependent variability of global gp10 shell structure. Variability of this type might be part of a proposed back-up motor [46] that is relatively simple from a physical perspective. The variability would eventually be lost when packaging was completed.

### 4.2. Interplay of N-Terminal Tails at Different Positions

One may envision at least two structural explanations for the observation of infrequent, yet fast, translocation events of A monomers (described in Section 3.2). First, it is possible that once the E monomer enters the cavity, the steric limitations are altered, resulting in nearly absent trans-shell displacements of the A monomer. Accordingly, if the vacant-pore rates of the E monomer are slightly faster than for the A monomer, there would be a greater likelihood that the E monomer will enter first and halt trans-shell displacement of the A monomer. This is a physically-intuitive rationalization, since translocation of the E-monomer tail will necessarily lead to a more constricted channel through which the A-monomer tail must pass.

A second explanation is that expansion leads to opening of the cavity, which introduces a time-dependent steric environment that differentially impacts the A monomer. In that scenario, the A-monomer tail could translocate during the initial stages of the simulation, though upon shell expansion, the cavity would disfavor trans-shell displacements of the A monomer. This type of asymmetry may be expected based on the structure around the A/E pore. Specifically, each A monomer is proximal to a penton, while the E monomer is proximal to a monomer of the hexon. Consistent with previous simulations that predict transient symmetry breaking during expansion [31], it is possible that similar transient asymmetries may contribute to differential N-terminal tail dynamics. While isolating the precise origins of asymmetry is beyond the scope of the current study, future variants of the model may be designed to distinguish between these potential contributors to tail dynamics.

### 4.3. The Future of Modeling Capsid Dynamics

With the ever-increasing capacity and accessibility of high-performance computing resources, combined with the development of novel theoretical models, the field of computational molecular biophysics is reaching a point where one can rapidly propose and test theoretical concepts pertaining to large assemblies. That is, while simulations of full capsids are still computationally demanding, it is becoming possible to iteratively introduce changes to the model and test the resulting dynamics. By starting with a simple energetic model, as done here, one may gradually increase the model complexity and carefully assess the effects of each additional factor.

In the case of T7, we are now ready to extend the single-basin structure-based model, in order to account for additional competing conformations. For example, one may introduce interactions that stabilize the capsid I, capsid II and mature-phage conformations. The relative stability of each conformation could then be modulated, in order to determine which aspects of maturation are robust to the energetic details. This general strategy has been widely used to study conformational rearrangements in multi-domain proteins [47], as well as in assemblies [29,33,48].

Thus, from a technical standpoint, the presented simulations provide a foundation, upon which future studies may explore the balance of intermediate populations and localized motions of individual monomers. An additional potential avenue of investigation would be to increase the model complexity by explicitly representing the interactions with DNA [49], which could separately treat the steric/entropic and electrostatic effects. Through this systematic extension of the models, and comparison with experiments, we anticipate that the rich features of phage maturation may be fully understood.

## Figures and Tables

**Table 1:** Total number of N-terminal tails observed to undergo trans-shell displacements. A total of 20 independent simulations were performed (1200 tails in total, per monomer position).

Monomer	Number of Events
A	10
B	70
C	112
D	658
E	845
F	117
G	144

## Supplementary Materials

The following are available online at https://www.mdpi.com/1999-4915/12/11/1273/s1, Video S1: Simulation of capsid expansion.
